# Supramodal Sentence Processing in the Human Brain: fMRI Evidence for the Influence of Syntactic Complexity in More Than 200 Participants

**DOI:** 10.1162/nol_a_00076

**Published:** 2022-09-29

**Authors:** Julia Uddén, Annika Hultén, Jan-Mathijs Schoffelen, Nietzsche Lam, Karin Harbusch, Antal van den Bosch, Gerard Kempen, Karl Magnus Petersson, Peter Hagoort

**Affiliations:** Max Planck Institute for Psycholinguistics, Nijmegen, the Netherlands; Donders Institute for Brain, Cognition and Behaviour, Centre for Cognitive Neuroimaging, Radboud University, Nijmegen, the Netherlands; Department of Linguistics, Stockholm University, Stockholm, Sweden; Department of Psychology, Stockholm University, Stockholm, Sweden; Department of Computer Science, University of Koblenz-Landau, Koblenz, Germany

**Keywords:** complexity, fMRI, sentence processing, supramodal, unification

## Abstract

This study investigated two questions. One is: To what degree is sentence processing beyond single words independent of the input modality (speech vs. reading)? The second question is: Which parts of the network recruited by both modalities is sensitive to syntactic complexity? These questions were investigated by having more than 200 participants read or listen to well-formed sentences or series of unconnected words. A largely left-hemisphere frontotemporoparietal network was found to be supramodal in nature, i.e., independent of input modality. In addition, the left inferior frontal gyrus (LIFG) and the left posterior middle temporal gyrus (LpMTG) were most clearly associated with left-branching complexity. The left anterior temporal lobe showed the greatest sensitivity to sentences that differed in right-branching complexity. Moreover, activity in LIFG and LpMTG increased from sentence onset to end, in parallel with an increase of the left-branching complexity. While LIFG, bilateral anterior temporal lobe, posterior MTG, and left inferior parietal lobe all contribute to the supramodal unification processes, the results suggest that these regions differ in their respective contributions to syntactic complexity related processing. The consequences of these findings for neurobiological models of language processing are discussed.

## INTRODUCTION

In order to extract meaning from the orthographic patterns or from the speech sounds, multiple processing steps are involved. One important step is to retrieve relevant word information from long-term memory (the mental lexicon; Altmann, 1998). This information includes the morphological makeup of words, their syntactic features, and lexical aspects of their meaning. But this is not enough. In many cases a simple concatenation of individual word meanings will not result in a correct interpretation (Jackendoff, 2002). The reason is that in language, words that belong together often do not go together (Lashley, 1951). This is what linguists refer to as non-adjacent dependencies between the lexical elements that make up an utterance. How to combine word information retrieved from memory into representations of sentence-level meaning that are constructed on the fly is what we refer to as *unification* (Hagoort, 2005, 2013; Hagoort & Indefrey, 2014; Vosse & Kempen, 2000, 2009). The number of non-adjacent elements that have to be kept online determines *unification complexity*. In this large functional magnetic resonance imaging (fMRI) study on sentence processing (*N* = 204), we address two outstanding questions. (i) To what extent is the network subserving unification operations independent of the modality of input (spoken and written)? This was investigated by confronting half of the participants with the materials in spoken format and half with the same materials in written format. (ii) Which nodes in the language network are modulated by variation in syntactic complexity? For each sentence presented to the participant, we calculated a measure of complexity, which allowed us to identify the areas that were most sensitive to complexity variations.

### Modality-Independence of Unification

It is generally assumed that at least some aspects of structure-building processes in the spoken and written modalities are subserved by similar modality-independent operations (Favier & Huettig, 2021). For instance, in the processing model of structure building called the *unification framework* (Vosse & Kempen, 2000, 2009), the attachment of each new lexical item to the incrementally constructed syntactic representation of the sentence is identical for both the visual and the auditory language input, but this has not been explicitly tested. In the *memory, unification, and control framework* (Hagoort, 2005, 2013), the word information mainly stored in the temporal lobe includes specifications of syntax, morphology, and information about word meaning (Joshi & Schabes, 1997; Vosse & Kempen, 2000). The process of unifying these lexical information types with the sentence and discourse context is constrained by lexical features in a process assumed to be *supramodal*. It has been suggested that after a forward sweep from sensory cortex to the left temporal and parietal lobe, top-down signals from the left inferior frontal gyrus (LIFG) re-enter the posterior regions in cycles of reactivation (Baggio & Hagoort, 2011), establishing a unification network with the involvement of at least two left hemisphere regions (left temporal/parietal cortex and LIFG, where the LIFG is the higher level node in the network). Visual and auditory language processing streams are hypothesized to converge on this supramodal unification network during comprehension, potentially starting in posterior areas (for a detailed account, see Wilson et al., 2018). The unification network thus includes both a frontal and a temporoparietal node, but the LIFG is thought to be crucial for the higher-level unification processes whereas the mental lexicon (or memory component) is thought to recruit especially the temporoparietal node.

### Sentence Complexity

The second aspect that we addressed is related to sentence complexity. Processing complexity in language processing is often due to the fact that words that belong together do not always go together; that is, they do not appear in adjacent positions. In a recent study (Futrell et al., 2015), it was found that there is an almost universal tendency (based on an analysis of 37 languages) to dis-prefer sentences in which structurally related words are far apart, presumably as a result of the extra processing costs associated with non-adjacency. Nevertheless, non-adjacency is a common phenomenon in language processing and a hallmark of human languages. It occurs not only in sentences with a left-branching structure, but also in sentences with a right-branching structure. There is evidence that on the whole, left-branching structures are harder to process than right-branching structures. An increased cost of maintenance and structure building for left-branching sentence aspects compared to right-branching sentence aspects was first suggested by Fodor and colleagues (Fodor et al., 1974). This claim of an added processing load for left-branching structures has been supported by evidence from both production and comprehension studies (Cheung & Kemper, 1992; Kemper, 1986, 1987; Norman et al., 1992).

Snijders et al. (2009) identified the LIFG and the middle temporal gyrus (MTG) as core unification regions in a study that contrasted sentence processing with the processing of word lists. In the current study, we used a similar paradigm but we extended it with an explicit manipulation and measure of sentence complexity.

The neuroimaging literature on sentence complexity is substantial (among many others, see Caplan et al., 1998; Cooke et al., 2002; Makuuchi et al., 2009; Meltzer et al., 2010; Peelle et al., 2004; Santi & Grodzinsky, 2010; Vogelzang et al., 2020), but most often the studies have been restricted to (a) comparing two conditions of complex vs. simpler sentences, and (b) measuring one sensory modality only (but see Braze et al., 2011; Constable et al., 2004; Michael et al., 2001; Shankweiler et al., 2008). When comparing two conditions of sentence complexity, most studies have realized this manipulation by comparing object-relative to subject-relative sentences, the former ones known to be more complex than the latter ones (e.g., Braze et al., 2011; Constable et al., 2004; Cooke et al., 2002; Meltzer et al., 2010; Michael et al., 2001; Peelle et al., 2004). In addition, some studies (e.g., Caplan et al., 1998; Makuuchi et al., 2009; Santi & Grodzinsky, 2010; Stromswold et al., 1996) compared more complex center embedded sentences to simpler right-branching sentences. In our study, a complexity measure is instead used to calculate the processing complexity for each individual sentence.

**Figure 1.**
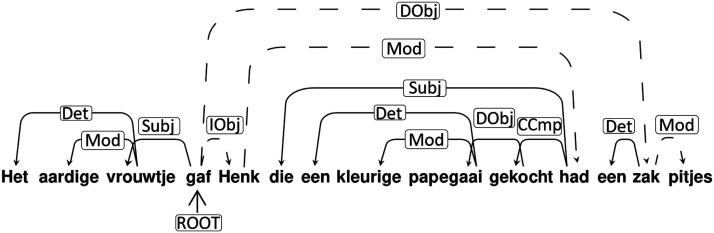
Left-branching dependencies point leftward from head to dependent. The left-branching processing complexity measure is calculated per sentence, as the maximum simultaneous non-resolved left-branching verbal dependencies (i.e., maximum number of dependents not yet assigned to a verb during the incremental parse). For the example sentence, this number equals 3 (reached after retrieval of the word *gekocht*). One of the open dependencies (from *papegaai*) is resolved after reading *gekocht* and other two at *had*, since the participle (*gekocht*) needs to be bound to an auxiliary (*had*), and the auxiliary needs to fill its subject argument with an antecedent that has the right number marking (i.e., *Henk*, singular). Included below the sentence are: a literal and non-literal translation into English, together with the corresponding word list with the translation. Labels on the arcs: Det: determiner; Mod: modifier; Subj: subject; Iobj: indirect object; Dobj: direct object; CCmp: complement clause.

Our complexity measure is motivated by two observations. (a) First is the finding that sentences with a left-branching structure (see Figure 1) are particularly hard to process (Cheung & Kemper, 1992; Kemper, 1986, 1987; Norman et al., 1992). We thus separated left-branching from right-branching complexity. (b) The second motivating observation is that there is a high processing cost related to building sentence structure with multiple simultaneous non-local dependencies. This is found for both natural (Makuuchi et al., 2009) and artificial grammars (Bahlmann et al., 2008; de Vries et al., 2012; Makuuchi et al., 2009; Uddén & Bahlmann, 2012; Uddén et al., 2012). Based on these two observations, our measure quantifies the amount of simultaneous left-branching non-local dependencies in a sentence (see Figure 1). It is of central importance that the sentence complexity is related to the incrementality of parsing from left to right.

To illustrate the importance of left to right processing, one could think of a *stack* (i.e., a first-in-last-out memory architecture) storing words that cannot yet be unified with the rest of the sentence structure. Each time a left-branching dependency is open, an element is pushed onto the stack, and only when it is closed is the element popped. The left-branching complexity measure thus corresponds to the stack depth used for a sentence. However, we do not want to make a strong assumption that there is a buffer (in the form of a stack or otherwise). If no buffering of words occurs during incremental processing, the complexity measure still singles out the sentences that have a high unification load for the following reason: Multiple simultaneously open dependencies lead to more options for the dependent to be unified with a head, when the head arrives. The number of simultaneously open dependencies is thus a complexity measure probing relevant syntactic processes independent of the existence of a stack or other buffers.

The left-branching complexity was of greatest interest in our neuroimaging study since we predicted that it would be associated with the greatest processing difficulties. Our left-branching complexity measure quantifies the maximum simultaneous number of dependents not yet assigned to the head of a *verb phrase*. Unification happens between the head of a phrase and its arguments. For instance, in a noun phrase the determiner and the adjective need to be unified with the noun as the head of the noun phrase (e.g., *The nice lady*; see Figure 1). The longer the distance between the heads and their arguments, and the more dependents and arguments there are to be maintained, the larger the processing demands (see Figure 1). In this case, verb phrases are especially relevant, since in general the verb is the nucleus of the proposition that is expressed in the sentence. For instance, upon reading the verb *kick*, a noun or the name of a person is expected to fill the argument slot for the agent of the action specified by the verb (e.g., *the man*). In addition, another noun is expected to take the argument slot for the undergoer of the action (e.g., *the ball*, as in *the man kicks the ball*). We expected processing to be more demanding if the arguments precede the head since the head is a stronger predictor for following arguments than the arguments are predictors for a following head. Therefore, we only count dependencies with a verbal head when we calculate the processing complexity. Although there are multiple ways to determine syntactic complexity, we note that in many cases these measures are highly correlated. (See Supplementary Results in the Supporting Information, which can be found at https://doi.org/10.1162/nol_a_00076.) Therefore, we surmise that our results are generalizable beyond the specifics of our choice for a measure of syntactic complexity.

To increase the sensitivity for unification associated with sentence complexity, we made sure that there was enough variance in the sentence structures. This was achieved by introducing relative clauses in half of our sentence material, while the other half had mixed sentence structures without relative clauses. Left-branching complexity was higher on average for sentences with relative clauses (see the Materials and Methods section). The analysis was, however, not a standard analysis comparing two complexity conditions, but a parametric analysis probing for the sentence complexity effect across all sentences in the experiment.

In addition to localizing a supramodal structure-building network in the brain, we also wanted to characterize the temporal dynamics of this network over the course of the sentence. While temporal dynamics are often studied using oscillatory modulations in electrocorticography (ECoG) or magnetoencephalography (MEG; Fedorenko et al., 2016; Lam et al., 2016), our large-scale fMRI study is a complement to this literature. In addition, testing only sentence average activations would decrease the sensitivity for potentially subtle modality-dependent effects of structure building. For this purpose, we divided the sentences into four time bins of equal length and formed linear contrasts to test increases and decreases in activity over the course of a sentence. For this analysis, we focused on predefined regions of interest (ROIs) found to be involved in structural unification (Snijders et al., 2009). Based on a recent meta-analysis of fMRI-studies of both syntactic and semantic sentence-level unification, the LIFG and the left posterior middle temporal gyrus (LpMTG) were chosen as primary ROIs (Hagoort & Indefrey, 2014; Tyler et al., 2010, 2013). The role of the left anterior temporal lobe (LaTL) in structure-building processes during sentence processing is less clear. So far, there is inconsistent evidence for the role of the aTL in syntactic processing. Some studies (e.g., Brennan et al., 2012; Rogalsky et al., 2008) report syntactic activations in the LaTL. However, many other studies have failed to find such activations. Moreover, other studies in patients with semantic dementia have found mainly semantic impairments (e.g., Chen et al., 2017; Lambon Ralph et al., 2012). Hence, the role of the aTL in syntactic processing is not yet fully substantiated or clear (cf. Bhattasali et al., 2019; Pallier et al., 2011). To investigate this further, we therefore also included a third ROI, based on coordinates from the study by Brennan et al. (2012), in a region in the LaTL.

## MATERIALS AND METHODS

### Participants

A total of 242 participants volunteered to participate in a larger study—the MOUS study (Mother of all Unification Studies; Schoffelen et al., 2019)—in which all participants took part in an fMRI and a MEG session. Of these, 38 participants were excluded (see next paragraph). The resulting 204 native Dutch speakers had a mean age of 22 years (range: 18–33 years). Half of the participants read sentences and word lists (visual group, 102 participants, 51 men), and the other half listened to auditory versions of the same materials (auditory group, 102 participants, 51 men). The study was approved by the local ethics committee (CMO; Committee on Research Involving Human Subjects in the Arnhem-Nijmegen region) and followed guidelines of the Helsinki declaration. All data of the MOUS study are available as open access with a full specification of the materials and design features, and shareable in the brain imaging data structure (BIDS) format (Schoffelen et al., 2019).

All participants were right-handed as assessed by the Bever (Bever et al., 1989) handedness questionnaire (including familial handedness), had normal or corrected-to-normal vision, and reported no history of neurological, developmental, or language deficits. We screened for medication use and excluded anyone on prescription medication. The instructions further contained a statement that no medication, alcohol, or drugs should be used on the day of the measurement. A total of 38 participants were excluded: (a) 20 because of technical problems (the most common were problems with data transfer, scanner hardware errors, presentation software problems resulting in faulty triggers, or absence of comprehension questions); (b) 4 because of poor data quality due to excessive blinking in the MEG session (affecting the amount of remaining MEG trials after artifact rejection), or more than 3.5 mm movement; (c) 6 because of study interruption; (d) 4 because participants did not fulfill the inclusion criteria (full list: 18–35 years, right-handed, self-reported Dutch monolingual language background, normal or corrected-to-normal vision, no self-reported history of neurological, developmental, or language deficits, MRI-compatibility (not pregnant, no claustrophobia, no incompatible devices, no incompatible tattoos, no incompatible self-reported operation history or metal in body)); and (e) 4 because of the participant’s poor compliance with the task as measured with the comprehension question performance (removal of these four outliers in task performance meant that all included participants had more than 59% correct, mean 80% correct +/− standard deviation of 9%; see further details in the Comprehension questions section).

### Language Stimuli

The stimuli consisted of 360 sentences and their 360 word list counterparts. Sentences were constructed to vary in complexity. One way to make complexity vary is by introducing a relative clause (Gibson, 1998). Half of the sentences contained a relative clause; the other half of the sentences were without a relative clause. Table 1 presents examples of the materials. The sentences varied between 9 and 15 words in length.

**Table 1.** Exemplar sentence and word list in Dutch and the literal English translation.

**Sentence**	**Word list**
Het aardige vrouwtje gaf Henk die een kleurige papegaai gekocht had een zak pitjes.	zak een kleurige aardige een had die vrouwtje papegaai gaf het gekocht pitjes
The nice lady gave Henk, who had bought a colorful parrot, a bag of seeds.	bag a colorful nice a had who lady parrot gave the bought seeds
Dit zijn geen regionale problemen zoals die op de Antillen.	zoals geen die Antillen problemen regionale zijn de dit op
These are not regional problems such as those on the Antilles.	such as not those Antilles problems regional are the these on

For each sentence, a corresponding word list was created by scrambling the words in the sentence so that three or more consecutive words did not form a coherent fragment. Since the sentence and word list conditions contain the same words, the comparison of sentences with word lists allowed us to probe the sentence-level unification process, while controlling for the retrieval of lexical items.

An important aspect of the syntactic complexity of the sentences can be formalized in terms of their dependency structure. The automatic FROG-parser (https://antalvandenbosch.ruhosting.nl/; https://languagemachines.github.io/frog/) was used to create a dependency tree for every sentence (see further details in the More on dependency trees section). The resulting trees were manually checked and corrected.

We calculated the complexity of each sentence on the basis of the dependency structure, separately for left- and right-branching sentence aspects. For the left-branching processing complexity measure, at each word we calculated the number of dependents that had not yet been attached to a verbal head (i.e., the head had not yet been encountered). The maximum number over the entire sentence was the left-branching complexity for that sentence. In other words, the left-branching complexity is the maximum number of simultaneously open left-branching dependencies (see Figure 1). Similarly, for right-branching complexity, at each word in each sentence, we calculated the number of dependents that would still connect to verbal heads that had been presented up to that word. The crucial difference between left- and right-branching constructions is the order of heads and arguments. In left-branching dependencies, the arguments precede the head. In right-branching sentences, the heads are followed by the arguments further downstream in the sentence. The calculation of the right-branching complexity measure is thus symmetric with respect to the left-branching complexity measure.

#### More on dependency trees: Heads, dependents, and the complexity measure

Like the more familiar phrase-structure trees, dependency trees encode syntactic aspects of word groups (phrases), but the aspects they emphasize differ. Phrase-structure trees specify the subphrases that a phrase is composed of, for instance, a verb phrase consisting of a verb followed by a noun phrase. Phrases typically include one word, called the Head, that is more important than the other subphrases, called dependents. In the example in Figure 1, the verb functions as the head because it imposes properties on the noun phrase rather than vice versa: It assigns accusative case to the noun phrase. Phrase-structure and dependency trees are similar because properties encoded by the former usually can be derived from the latter, and vice versa. We have opted for dependency trees for a practical reason—the availability of a high-quality dependency parser for Dutch.

Relative clauses are often ambiguous between a *restrictive* and a *non-restrictive*/*appositive* semantic interpretation. However, we wish to note that these differing interpretations are not associated with differing syntactic structures (see the analysis in Arnold, 2004, p. 39ff, which applies to Dutch as well). This means that the parse tree computed by the participants does not depend on the interpretation they choose. The complexity measure is thus not affected by this ambiguity. We estimate that in at most five sentences in the materials, the relative clauses needed a restrictive interpretation in order to make sense. In the written sentences, we did not use commas to separate the relative clauses from their antecedents, thus leaving the interpretation to the preference of the participants. (In Dutch spelling, such commas are optional, like in English; but unlike in German, where they are obligatory, irrespective of interpretation. In the spoken sentences, the sentences were pronounced with a prosodic pattern that was neutral between the interpretations.) In order to calculate the right-branching sentence complexity measure, we took the maximum of the word-by-word right-branching complexity as the right-branching complexity of that sentence. Thus, in other words, the right-branching complexity measure is the maximum number of simultaneously open right-branching dependencies.

For both left- and right-branching complexity calculations, we focused on verbal heads since they head clauses. Clausal structure is more encompassing than the more local structure of phrases headed by other parts of speech (e.g., noun phrases, prepositional phrases, adjectival phrases). To verify this for our own material, we compared the left-branching complexity between sentences, with and without relative clauses, for verbal and non-verbal heads. For verbal heads, left-branching complexity was higher for sentences with relative clauses (mean = 3.0 +/− 0.7 standard deviations) than sentences without (mean = 1.4 +/− 0.9 standard deviations). For non-verbal heads, there was no significant difference between sentences with relative clauses (mean = 1.9 +/− 0.5 standard deviations) and those without (mean = 2.1 +/− 0.5 standard deviations). It is assumed that clausal structure exerts the main influence on cognitive complexity. Note that although our complexity measures are designed to index *processing* complexity, for the sake of brevity we will refer to the outcome of calculations using these measures as *left*/*right-branching complexity*.

### Task and Procedure

Within a measurement session, the stimuli were presented in a mini-block design, and alternated between a sentence block (5 sentences) and a word list block (5 word lists), for a total of 24 blocks. The type of starting block (sentences or word lists) was counterbalanced across subjects.

#### Comprehension questions

In order to check for compliance, 10% of the trials were followed by a *yes*/*no* question about the content of the just-presented sentence/word list. Half of the questions on the sentences addressed the content of the sentence (e.g., *Did grandma give a cookie to the girl?*) whereas the other half, and all of the questions on the word lists, addressed one of the main content words (e.g., *Was the word ‘grandma’ mentioned?*). Half of the questions on complex relative clause sentences concerned the content from the relative clause, to make sure the participants comprehended all parts of the sentence. Subjects answered the question by pressing a button for “Yes”/“No” with their left index and middle finger, respectively. Based on the answers to the catch trial comprehension questions, subjects were defined as outliers (i.e., subjects with a negative distance >1.5 times the interquartile range, from the mean) and were excluded from further analysis, as not compliant with the task. This procedure resulted in a threshold of 59% percent correct, which all 204 included subjects passed (mean 80% correct +/− standard deviation of 9%).

#### Stimulus presentation

For visual stimulus presentation, sentences/word lists were presented word-by-word with a mean duration of 351 ms for each word (minimum of 300 ms and maximum of 1,400 ms, depending on word length, etc., see further final paragraph of this section). Corresponding visual sentences and word lists had the same total duration. The median duration of whole sentences/word lists was 8.3 s (range 6.2–12 s). Auditory sentences had a median duration of 4.2 s (range 2.8–6.0 s), and were spoken at a natural pace. The matching word list words were also read at a natural pace, with a brief pause between words, averaging 7.7 s (range 5.5–11.1 s) per word list.

Each participant was presented with 60 sentences and 60 word lists, either entirely in the visual or the auditory modality. The full stimulus material consisted of 180 sentences and 180 word lists, divided into three subsets such that each participant saw 2/3 of the stimuli set in the MEG session (120 trials of each condition) and 1/3 in the fMRI session (60 trials). Across participants, each subset was presented as many times in MEG as in fMRI.

Each participant was presented with each stimulus once, in either the sentence or the word list condition, but not in both. The presentation of the sentence and word list versions of the items was counterbalanced across subjects. The auditory versions of the stimuli were recorded by a female native Dutch speaker. The word lists were pronounced with a neutral (i.e., flat) prosody and a clear pause (at least 100 ms for the auditory word lists and around 300 ms for the visual word lists) between each word.

To indicate sentence and word list blocks, the start of each block began with a 1,500 ms block type indication, *zinnen* ‘sentences’ or *woorden* ‘words’. Only in sentences did the first word begin with a capital letter, while the last word ended with a full stop. The intertrial interval was jittered between 3,200–4,200 ms. During this period, an empty screen was presented, followed by a fixation cross.

The visual stimuli were presented with a LCD projector (vertical refresh rate of 60 Hz) situated outside the fMRI scanning room, and projected via mirrors onto the screen inside the measurement room. All stimuli were presented in a black monospaced font on a gray background at the center of the screen within a visual angle of 4 degrees using Presentation software (Version 16.0, Neurobehavioral Systems, Inc.; https://www.neurobs.com/). For auditory stimulus presentation, sounds were presented via plastic tubes and ear pieces to both ears. Before the experiment, the hearing threshold was determined individually, and the stimuli were then presented at an intensity of 50 dB above the hearing threshold, with the obtained volume individually pretested on top of the echo planar imaging (EPI) sequence noise, to verify that all stimuli were clearly audible.

Each word was separated by an empty screen for 300 ms before the onset of the next word. The presentation time of each word was weighted by the number of letters in the word, providing a natural reading experience (c.f. Nieuwland & Van Berkum, 2006). In this way reading of the words was also matched to some extent to the total duration of the audio version of the stimuli. For any given sentence (or word list) the variable presentation duration of a single word was a function of the following quantities: (i) the total duration of the audio version of the sentence/word list (audiodur), (ii) the number of words in the sentence (nwords), (iii) the number of letters per word (nletters), and (iv) the total number of letters in the sentence (sumnletters). Specifically, the duration (in ms) of a single word was defined as: (nletters/sumnletters) * (audiodur + 2000 − 150 * nwords). It is reasonable to include both number of words and number of letters in this formula for adaptive rapid serial visual presentation, as previous research found decreased task load ratings for adaptive compared to fixed rapid serial visual presentation (i.e., with fixed presentation time for each word), using both parameters (Öquist & Goldstein, 2003). The minimum duration of short words was set to 300 ms irrespective of the relative weighting described by the formula. While there are limitations to this method, as it leaves out multiple factors known to affect reading time (e.g., word frequency), it is an adequate approximation suitable for languages with fairly regular grapheme–phoneme correspondences such as Dutch (as opposed to, e.g., English). Note that this formula does not apply to the auditory sentences, which were spoken at a natural pace.

### MRI Data Acquisition

The data were acquired with a SIEMENS Trio 3T scanner using a 32-channel head coil. We used a whole head T2*-weighted echo-planar blood oxygenation level-dependent (EPI-BOLD) sequence. The single-echo 2D ascending slice acquisition sequence (partial brain coverage) had the following specifications: Volume TR = 2.00 s, TE = 35 ms, 90 degree flip-angle, 29 oblique slices, slice-matrix size = 64 × 64, slice thickness = 3.0 mm, slice gap 0.5 mm, FOV = 224 mm, voxel size (3.5 × 3.5 × 3.0 mm^3^ during acquisition; interpolated to 2 × 2 × 2 mm^3^ in SPM). The number of volumes acquired per participant was around 650 for the auditory group and around 850 volumes for the visual group. The exact number of volumes varies slightly (e.g., 10 volumes) per participant due to different response times to the comprehension questions.

For the structural MR image volume, a high-resolution T1-weighted magnetization-prepared rapid gradient-echo pulse sequence was used (MP-RAGE; TE = 3.03 ms, 8 degree flip-angle, 1 slab, slice-matrix size = 256 × 256, slice thickness = 1 mm, FOV = 256 mm, isotropic voxel-size = 1.0 × 1.0 × 1.0 mm^3^).

### Preprocessing

The primary imaging data were checked for quality, including checks of subject movement and signal drop out. The data were preprocessed with statistical parametric mapping software (SPM8; Welcome Trust Centre for Neuroimaging, London, UK; www.fil.ion.ucl.ac.uk/spm), which was also used for statistical analysis at the first- and second-level. Coordinates are reported in Montreal Neurological Institute (MNI) space throughout.

The first three EPI-BOLD volumes were removed to ensure T1-equilibrium. The remaining volumes were (1) realigned to correct for individual subject movement and (2) corrected for differences in slice-acquisition time.

The mean EPI-BOLD volumes were co-registered to the structural image (i.e., the structural image was the reference and the mean EPI-BOLD volume was the source image), and this transformation was then applied to all EPI-BOLD volumes. Structural images were spatially normalized to the structural image (T1) template provided by SPM8, using affine regularization. The transformation matrices generated by the normalization algorithm were applied to the corresponding functional EPI-BOLD volumes. All structural and functional images were spatially filtered with an isotropic 3D spatial Gaussian filter kernel (full width at half maximum; FWHM = 10 mm).

### Statistical Analysis

The fMRI data were analyzed statistically, using the general linear model framework and statistical parametric mapping (Friston et al., 2007) in a two-step mixed-effects summary-statistics procedure.

#### First-level models

The single-subject fixed effect analyses included a temporal high-pass filter (cycle cut-off at 128 s) to account for various low-frequency effects. All linear models included the six realignment parameters from the movement correction. All models included two regressors modeling the sentence and word list conditions from the onset of the stimulus throughout the duration of its presentation and the following three regressors: the intertrial interval, the interblock interval, as well as the comprehension questions. This minimal model (a part of all models) was used to probe the language/sentence processing network (compared to a low-level baseline, more specifically the interblock interval) as a localization of language relevant regions. Intertrial and interblock intervals were modeled separately as we wanted to use the interblock intervals as a low-level baseline potentially less affected by lingering processing of the stimulus after its presentation. Comprehension questions were modeled from the onset of the question until after the reply (hence including the reply). Additional models (including additional regressors) as well as contrasts are described in more detail below.

##### Left- vs. right-branching complexity.

Regressors containing two sets (corresponding to sentences and word lists, respectively) of five parametric sentence/word list modulators, included: (1) number of letters; (2) number of syllables; (3) number of words; all of them referred to as *control regressors*; (4) the right-branching complexity; and (5) the left-branching complexity. The timing of these modulators does not differ from the timing of the sentences/word lists they modulate (i.e., from the onset of the stimulus throughout the duration of its presentation). All of the regressors were used both for the auditory and visual analyses. These parametric modulators were entered as user-specified regressors after convolution with the canonical hemodynamic response function (HRF). To control for lexical level features, we associated each scrambled word list version of a sentence with the left- and right-complexity measure of that sentence and contrasted complexity for sentences and word lists (using the contrasts [0 0 0 0 0 0 0 0 1 0 0 0 0 −1 0]; [0 0 0 0 0 0 0 0 0 1 0 0 0 0 −1]; where the first five columns are described in the First-level models section, the next five columns are the modulators of sentences, and the last five are modulators of word lists). To verify the expectation that complexity should affect sentences and not unstructured word lists, we also created contrasts probing the sentences and word lists separately, against the implicit baseline (using the contrasts [1 0 0 −1]; [0 1 0 −1]). We also formed the reverse contrasts (word lists > sentences, i.e., negative effects of complexity) to those described in this paragraph.

##### Left-branching processing complexity: Temporal dynamics.

In order to analyze activation changes over sentences, we performed two analyses: One was a contrast analysis with four time bins and one a finite impulse response (FIR) analysis with seven time points. Note that there is no exact correspondence between words or other structures and time bins/time points. We used the complexity measures calculated on the whole sentence level in these analyses.

The first analysis divided each sentence/word list presentation into four equally long time bins. For the time-bin analysis, parametric modulators were specified using pmod (parametric modulation) functionality implemented in SPM8. The order in which regressors are entered matters for this analysis, since each pmod regressor is orthogonalized with respect to the so called *unmodulated* regressors, as well as earlier pmod regressors (Mumford et al., 2015). Control regressors (i.e., numbers of words, letters, and syllables) were thus entered before the regressors encoding the complexity measures, and the right-branching complexity measure was entered before the left-branching complexity measure, when assessing the effects of left-branching complexity and, conversely, when assessing the effects of right-branching complexity.

We focused on the two ROIs that were most clearly and selectively responsive to left-branching complexity: the LIFG and LpMTG. To assess increases and decreases over the course of the sentences, we formed first-level linear contrasts across the complexity regressors corresponding to each time bin (e.g., [−2 −1 1 2]).

In the second analysis, time courses were extracted from the LIFG and LpMTG ROIs using the FIR model as implemented in MarsBar (Brett et al., 2002). The FIR model does not assume any particular shape of the hemodynamic response, but estimates the signal at each time bin (henceforth called *time windows*) separated by one TR (in our case 2 s), up to 24 s after stimulus onset, adjusting for effects of stimuli overlapping in time. As the naturally spoken sentences created shorter auditory presentation times, we used the visual sample in this analysis. Since our average visual stimulus duration was 8.3 s and our average intertrial interval was 3.7 s, we analyzed the first seven time windows, covering 0–14 s post stimulus onset. We analyzed the differences in percent signal change between high (left-branching complexity >=3) and low (left-branching complexity <3) complexity, by introducing two sets of regressors (high/low) for sentences and word lists in the FIR model. The differences in percent signal change for high–low complexity were tested with a paired *t* test at all time points. We corrected for multiple comparisons over seven time points using Bonferroni correction, resulting in a corrected alpha level of 0.05/7 = 0.007. In a follow-up analysis, we tested whether the complexity effects came in different time windows for LpMTG, compared to LIFG. We compared the difference scores of the percent signal change for high complexity sentences – low complexity sentences, for LpMTG-LIFG, using a paired *t* test.

#### Second-level statistics and visualization

The second level comparison used a two-sample unequal-variance *t* test. Visual and auditory data were modeled separately and then compared using an additional model. Five such models were created: One of each is reported in the first three sections of the Results and two models (where one was a FIR model) in the fourth section of the Results. (See the supplement for additional results.) Conjunctions were tested against the conjunction null (Nichols et al., 2005). As we used the conservative version of conjunction analysis, we are only observing voxels/regions where the null hypothesis is rejected in both modalities. Throughout the article, statistics are reported with the following criterion for significance: presence of family-wise error (FWE) corrected voxels or clusters at *p*_FWE_ < 0.05, both in the whole brain analysis and in the ROI analysis, using a threshold of *p* < 0.005 uncorrected. In other words, while significant clusters and peak voxels often coincide, we report clusters even if there were no significant peak voxels in them, and we report a peak voxel even if it would be outside of a cluster. In tables, all clusters significant at *p*_FWE_ < 0.05 on the whole brain level are reported including locations of voxels belonging to a cluster. Location and statistics of significant peak voxels (within or outside significant clusters) are also reported.

#### ROI analysis

In the ROI analysis, we used 10 mm spherical ROIs around meta-analytically established activation hotspots of semantic and syntactic unification, reported in Talairach space (Hagoort & Indefrey, 2014). The two top coordinates for semantic and syntactic unification were close to each other, one pair in the LIFG and another in the LpMTG. We used the mean coordinate between the syntactic and semantic peak for our ROI analysis. This resulted in the (Talairach space) coordinates [*x* *y* *z*] = [−45 20 10] for LIFG and [−50 −45 5] for LpMTG, which we then transformed to MNI space using the Talairach Daemon (https://www.talairach.org/).

In addition, a LaTL ROI was investigated in the left- vs. right-branching complexity. This was again a 10 mm spherical ROI around the peak coordinate reported for the building of sentence structure in a related study (Brennan et al., 2012). These were [*x* *y* *z*] = [−50 11 −16] in MNI space.

#### Anatomical inference

All local maxima are reported as MNI coordinates. Assignment of clusters and voxels to gyri, sulci, and Brodmann areas (for LIFG) were done using the Anatomy toolbox (Version 21) implemented in SPM (Eickhoff et al., 2005).

## RESULTS

### Sentence Processing vs. Low-Level Baseline

We here present the second-level analysis of the per-subject generated first-level contrast.

**Figure 2.**
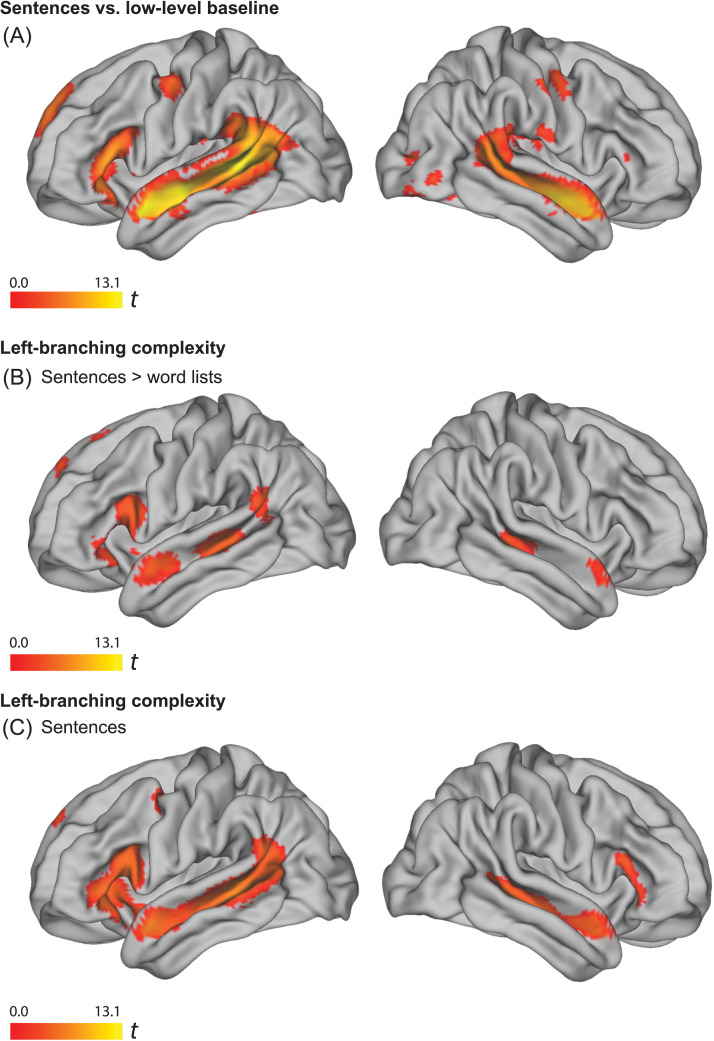
Results images were created based on the conjunction of data of the visual (*N* = 102) and the auditory (*N* = 102) presentation of the materials, using a threshold of *p* < 0.005 uncorrected, for the contrasts with significant results at the cluster or voxel level, using a criterion of *p*_FWE_ < 0.05. (A) Effect of sentences > low-level baseline. (B) Parametric effect of left-branching complexity in sentences > word lists (word list complexity is the complexity of the corresponding sentence). Peaks in a significant temporal cluster were observed in LpMTG and LaTL. An LIFG ROI contained a significant cluster. (C) Parametric effect of left-branching complexity in sentences (i.e., without comparing word lists). Peaks in a large significant frontotemporal cluster, were observed in LpMTG, LaTL, and LIFG. LpMTG: left posterior middle temporal gyrus; LaTL: left anterior temporal lobe; LIFG: left inferior frontal gyrus.

We verified that our paradigm activated the classical perisylvian language network by comparing activation during sentence processing with a low-level baseline (i.e., fixation/rest interblock interval). A significant cluster spanning left anterior to posterior superior temporal cortices (middle and superior temporal gyrus), left inferior frontal gyrus (BA 44/45), the left inferior parietal lobe (LIPL), and left fusiform gyrus was observed (see Figure 2A). Two additional clusters (right superior temporal and right fusiform gyrus) as well as significant voxels in left orbitofrontal cortex and in left precentral and left superior frontal gyrus were also observed.

### Left-Branching Complexity

To investigate supramodal unification (Hagoort, 2005; Vosse & Kempen, 2000), we created conjunctions over visual and auditory contrasts of parametrically varied sentence complexity. We controlled for lexical features correlated with sentence complexity by subtracting corresponding word list parameters in the following way. Each scrambled word list version of a sentence was associated with the left- and right-branching complexity measure of that sentence. We analyzed the parametric effects of left-branching complexity for sentences compared to the corresponding effects for word lists (henceforth referred to as *sentences* > *word lists contrasts*).

There was a significant interaction between the parametric effect of left-branching complexity in sentences compared to word lists throughout the left perisylvian language network (Figure 2B and Table 2). In this comparison, a significant cluster was observed in posterior middle/superior temporal cortices, anterior superior temporal gyrus (anterior temporal lobe), left inferior frontal gyrus (BA 44 and 45), and left inferior parietal cortex. There were no significant effects in the reverse comparison, word lists > sentences. To consolidate the findings in the sentences > word lists contrast, we verified that complexity had no effect on word lists by investigating the effect of left-branching processing load in sentences and word lists separately. There were no significant effects of complexity in the word list condition. In contrast, we observed a positive effect of left-branching complexity for sentences in the left perisylvian language network (Figure 2C and Table 2). There was no significant activity in the reverse comparison.

**Table 2.** Supramodal (parametric) effect of left-branching complexity.

Region	Cluster size	Cluster *p*_FWE_	MNI coordinates			Voxel *p*_FWE_	Voxel *t*_201_
			*x*	*y*	*z*		
Left-branching*							
Sentences > words							
*LpMTG/LaTL/LIFG/LIPL*	2,678	0.001					
*LpMTG/STS*			−52	−30	−4	0.003	5.13
*LaTL* + *LIFG (BA 44,45), LIPL*			−56	2	−16	0.042	4.47

Left-branching							
Sentences							
*LpMTG/LaTL/LIFG/LIPL*	4,225	<0.001					
*LpMTG/STS*			−50	−36	−2	<0.001	7.67
*LaTL*			−54	8	−18	<0.001	6.40
*LIFG (BA45)*			−48	28	0	0.001	5.19
*LIFG (BA44)*			−56	18	14	0.003	5.15
*LIPL I*			−50	−54	16	0.011	4.82
*LIPL II*			−54	−56	18	0.013	4.77
*LIPL III*			−52	−54	12	0.019	4.68

Right-branching							
Sentences							
*ROI LaTL, 10 mm, SVC*	37	0.048					
			−54	8	−20	0.024	3.13

*Note*. No significant activations (at either ROI level) for the opposite contrasts, i.e., Left-branching, Words > Sentences; negative effect of Left-branching, Sentences. LpMTG: left posterior middle temporal gyrus; LaTL: left anterior temporal lobe; LIFG: left inferior frontal gyrus; LIPL: left inferior parietal lobe; STS: superior temporal sulcus; ROI: region of interest; SVC: small volume correction; FWE: family-wise error; MNI: Montreal Neurological Institute.

**Figure 3.**
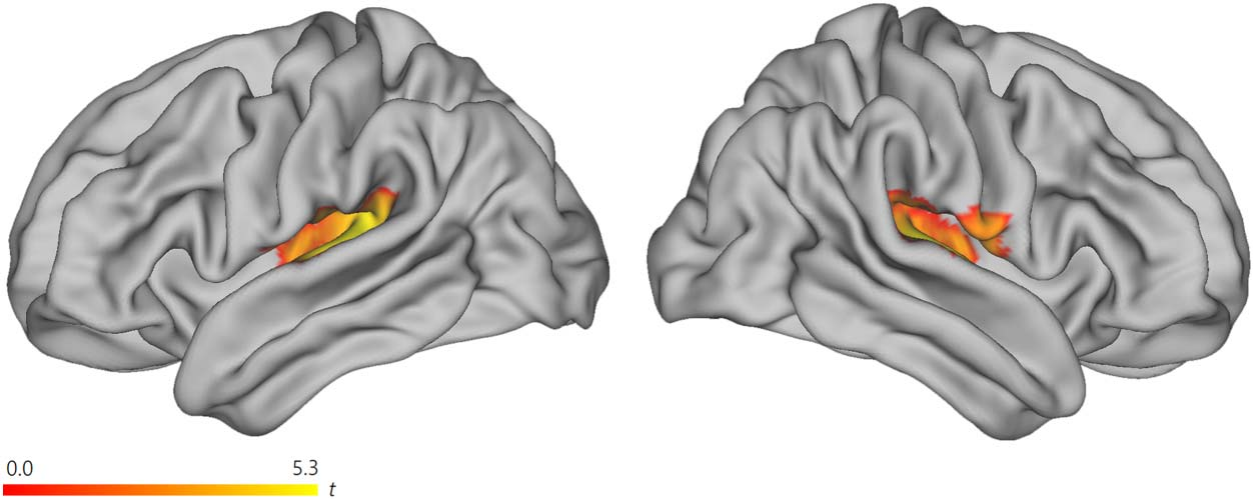
Visual > auditory effect for the left-branching complexity measure, for sentences. There was generally an absence of modality-specific effects across almost all contrasts reported, with this contrast as one of the exceptions. In a follow-up analysis, we tested whether the observed effects in this visual > auditory contrast was due to an effect of left-branching complexity in the opposite/negative direction (i.e., higher BOLD response for lower complexity) for the auditory group. This was the case (see Table 3).

Across all contrasts, the number of modality-specific aspects were minimal. There were no significant effects for visual > auditory or auditory > visual contrasts for left-branching complexity in the sentences > word lists comparison. There were no significant activations for auditory > visual for the left-branching complexity measure for sentences. There were, however, visual > auditory effects for the left-branching complexity measure for sentences (Figure 3, Table 3). There were clusters observed in the bilateral superior temporal gyrus (STG) / Heschl’s gyrus and left subiculum. In a follow-up analysis, we tested whether the observed effects in this visual > auditory contrast was due to an effect of left-branching complexity in the opposite/negative direction (i.e., a higher BOLD response for lower complexity) for the auditory group. This was the case (see Table 3).

**Table 3.** Modality specific effects left-branching complexity.

Region	Cluster size	Cluster *p*_FWE_	MNI coordinates			Voxel *p*_FWE_	Voxel *t*_201_
			*x*	*y*	*z*		
Left-branching							
Sentences							
Visual > auditory							
*LSTG/Heschl’s gyrus*	1,575	0.011					
			48	−16	8	<0.001	5.74
			44	−24	10	0.001	5.44
*RSTG/Heschl’s gyrus*	1,764	0.009					
			−38	30	12	<0.001	5.73
*Left subiculum*	1,221	0.031					

Right-branching							
Sentences > word lists							
Auditory > visual							
*ROI LIFG 10 mm, SVC*	36	0.048	−52	20	10	0.003	2.96

Left-branching							
Negative direction							
Sentences							
Auditory							
*LSTG*	1,988	0.004					
			−38	−30	12	<0.001	6.16
			−48	−20	8	<0.001	5.81
*RSTG*	1,741	0.007					
			50	−16	8	<0.001	6.10
*Left fusiform gyrus*			−32	−38	−10	0.009	4.87

*Note*. No significant activations for visual > auditory or auditory > visual in: Left, Sentences > word lists; Right, Sentences. No significant activations for auditory > visual in: Left, Sentences. No significant activations for visual > auditory in: Right, Sentences > word lists. LSTG: left superior temporal gyrus; RSTG: right superior temporal gyrus; LIFG: left inferior frontal gyrus; ROI: region of interest; LIPL: left inferior parietal lobe; SVC: small volume correction; FWE: family-wise error; MNI: Montreal Neurological Institute.

### Effects of Right-Branching Complexity

For the corresponding comparisons for right-branching complexity to those reported in the Language Stimuli section, no significant effects were found for sentences > word lists nor for word lists > sentences. For the separate sentence contrast, there were no significant voxels or clusters. Our ROI analysis included three ROIs (LIFG, LpMTG, and LaTL). The LaTL ROI contained a significant cluster for an increase in right-branching complexity. No significant effects were observed for word lists nor for the corresponding comparisons in the reverse direction.

There were no significant effects for visual > auditory or auditory > visual contrasts for the right-branching complexity measure for sentences. There were no significant activations for visual > auditory for right-branching complexity in the sentences > word lists comparison.

### Left-Branching Complexity: Temporal Dynamics

Unification processes operate incrementally during word-by-word presentation of sentences. The unification load will thus vary over time, in particular for complex sentences. To describe the temporal dynamics of sentence unification, we divided each sentence and word list into four time bins of equal length. We focused on the left-branching complexity measure, since it showed the most prominent effects. For the ROI analysis, we used the two ROIs that were most clearly active specifically to left-branching complexity: the LIFG and LpMTG. The right-branching complexity parameter and control parameters (number of words, number of syllables, and number of letters) were entered into the model to ensure that we were studying the contribution of left-branching complexity to dynamic changes over time. We created linear contrasts probing monotonic increases and decreases in the parametric effect of the complexity measures across the four time bins. We expected temporal dynamics related to sentence unification to be stronger in sentences compared to word lists.

**Figure 4.**
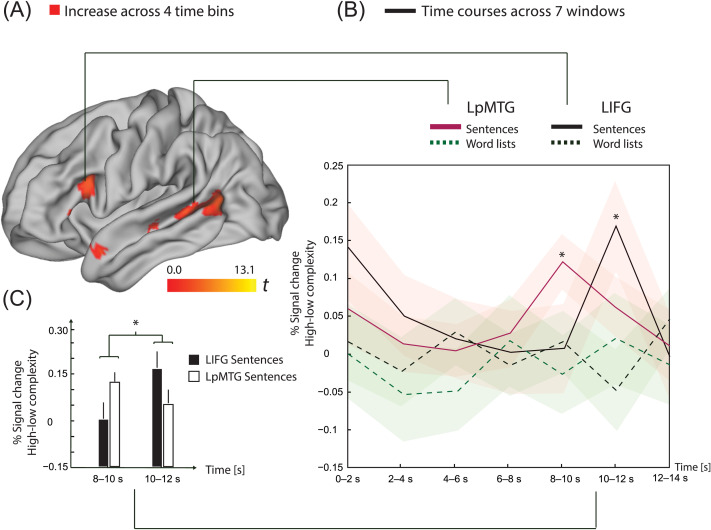
(A) In red, left-branching complexity: increase across four time bins for sentences. Results images were created based on the conjunction of the data for visual and auditory presentations of the materials, using a threshold of *p* < 0.005 uncorrected, for the contrasts with significant results at the cluster or voxel level, using a criterion of *p*_FWE_ < 0.05. In the LpMTG ROI, there was a significantly (using SVC) steeper increase for sentences > word lists. (B) To illustrate the observed dynamics in (A) further, with time courses using a FIR model, we focused on the written sentences and word lists which have longer presentation times for sentences (compared to the naturally spoken sentences) and thus provides more robust estimates of changes in the BOLD response over the sentence. Complexity difference scores (high–low) of % signal change from LIFG and LpMTG ROIs, in sentences and word lists are plotted over seven time windows. Note that we are thus contrasting sentences against sentences and word lists against word lists (not sentences against word lists). Red shaded areas represent +/− 1 standard error of the mean around each data point for sentences and likewise for word lists, in green shade. The % signal change is relative to the mean activity of this ROI in the whole session; thus, note that the first time point (0–2 s) can be active since a few words were already presented during this time. The complexity effect within sentences was significant at 8–10 s in LpMTG (*t*_101_ = 3.28, *p*_Bonf_ = 0.01) and at 10–12 s in LIFG (*t*_101_ = 2.82, *p*_Bonf_ = 0.04). Both of these survive Bonferroni correction for testing seven time points. The seventh time point (12–14 s) contains the intertrial interval, on average. All four mean time courses returned to the session mean at this time point. (C) The complexity effect for sentences occurred in an earlier time window for LpMTG, compared to LIFG. There was a significant interaction between the sentence complexity effect in LpMTG vs. LIFG, at 8–10 s vs. 10–12 s (*t*_101_ = 2.39, *p* = 0.02). The data in (C) are the same as in the corresponding time windows in (B). LpMTG: left posterior middle temporal gyrus; LIFG: left inferior frontal gyrus; FWE: family-wise error; ROI: region of interest; SVC: small volume correction; FIR: finite impulse response; BOLD: blood oxygen level-dependent.

As shown in Figure 4A and Table 4, we observed a monotonic increase related to sentence unification in the LpMTG ROI. When analyzing sentences and word lists separately, a monotonic increase over sentences was observed in both the LIFG and LpMTG ROIs. No decrease was observed. There were no effects of modality (visual > auditory or auditory > visual) for the contrasts reported in this section.

**Table 4.** Supramodal (parametric) effect of left-branching complexity, increase across 4 time bins.

Region	Cluster size	Cluster *p*_FWE_	MNI coordinates			Voxel *p*_FWE_	Voxel *t*_201_
			*x*	*y*	*z*		
Left-branching							
Sentences > words							
*ROI LpMTG 10 mm, SVC*	74	0.034					
			−46	−42	0	0.025	3.29

Left-branching							
Sentences							
*ROI LpMTG 10 mm, SVC*	242	0.029	no significant peak voxels				
*ROI LIFG 10 mm, SVC*			−50	20	16	0.028	3.25

*Note*. No significant activations (at either ROI level) for: any of the corresponding decreases; Right, Sentences > words; Right, Sentences. Furthermore, there were no effects of modality (visual > auditory or auditory > visual). ROI: region of interest; LpMTG: left posterior middle temporal gyrus; SVC: small volume correction; LIFG: left inferior frontal gyrus; FWE: family-wise error; MNI: Montreal Neurological Institute.

To follow up on this finding, we extracted percent signal change time courses (using a FIR model) from the LIFG and LpMTG ROIs in the reading group (Figure 4B). The visual sample has longer presentation times compared to the naturally spoken sentences. Thus, more robust estimates of changes in the BOLD response over the sentence are expected for this part of the sample. As the finite impulse response model allows the extraction of time courses from ROIs and does not assume any particular shape of the hemodynamic response, this was an appropriate follow-up to investigate any potential differences in time courses between the ROIs and to characterize the time courses in greater detail. We analyzed the differences in percent (%) signal change between high (left-branching complexity >=3, 168 sentences) and low (left-branching complexity <3, 192 sentences) complexity as a function of time. The complexity effect (high–low) for sentences was significant at 8–10 s in the LpMTG ROI (*t*_101_ = 3.28, *p*_Bonf_ = 0.01) and at 10–12 s in the LIFG ROI (*t*_101_ = 2.82, *p*_Bonf_ = 0.04). We Bonferroni-corrected for testing seven time points. The complexity effect for sentences thus occurred in an earlier time window in LpMTG than in LIFG. There was a significant difference between the sentence complexity effect in LpMTG–LIFG, at 8–10 s vs. 10–12 s (*t*_101_ = 2.39, *p* = 0.02, see Figure 4C). The seventh time point (12–14 s) contains the intertrial interval.

## DISCUSSION

Our study provides support for the hypothesis that unification should be understood as a supramodal process, common to sentences presented as speech or text. The current results show that the supramodal network subserving unification includes the LIFG, the bilateral MTG (both anterior and posterior parts), as well as LIPL. We observed an effect of left-branching complexity in the perisylvian language regions, irrespective of modality. Moreover, we observed an absence of modality specific effects related to unification processes (i.e., no effects when comparing visual > auditory or auditory > visual samples). This finding provides further support for unification as a process that is not modality specific. Our results thus support a view where the visual and auditory input streams converge in a supramodal unification network, most clearly in LIFG and LpMTG.

Previous studies comparing complex vs. simple sentences have implicated the inferior frontal and temporal heteromodal association cortices as supramodal regions associated with sentence structure manipulations (Braze et al., 2011; Constable et al., 2004; Michael et al., 2001; Shankweiler et al., 2008). Our large sample size and the use of parametrical variation of processing complexity increase both sensitivity and validity compared to these previous studies. Our results support the view that LIFG and the bilateral MTG (both anterior and posterior parts) are key nodes of the supramodal unification network. Moreover, our results suggest that additional regions (e.g., LIPL; see further discussion below) could be considered a part of this network (Binder et al., 2009). These regions correspond well to the overlap of written and spoken comprehension studies on sentences reported in a recent meta-analysis study (Walenski et al., 2019) and in a study using contrasts of speech vs. backwards speech and written sentences vs. scrambled written sentences (Wilson et al., 2018). The results are largely in agreement with the findings reported by Friederici and colleagues (for an overview of these findings, see Friederici, 2017), who also found complexity effects in both LIFG and posterior temporal cortex. In addition, Friederici (2017) refers to activations in anterior STG as related to local structure building, which presumably refers to the structure building at the phrasal level that others (Snijders et al., 2009) have found to be related to LpMTG.

The effects of left-branching complexity show increased activation levels in mainly left perisylvian language regions for more syntactically complex sentences. Moreover, the results show that the effect of left-branching processing complexity in LIFG and LpMTG increased toward and even beyond the end of the sentence. The corresponding changes were nonsignificant for LIPL, and similar sentence progression effects were not observed for the right-branching structures. Thus, in summary, left-branching complexity effects are stronger and show different dynamics over the sentence compared to right-branching complexity effects. This highlights the fact that the neuronal underpinnings of sentence processing operations cannot be fully appreciated without understanding how a complexity measure indexes the sentence processing operations. Another good example of this approach can be found in a recent ECoG study on visual sentence processing (Nelson et al., 2017; see also Fedorenko et al., 2016). By using many processing models to predict neural activity over the sentence, they found evidence for a structure building process in LIFG and posterior superior temporal cortices. It is possible that a substantial part of the variance in the neural dynamics explained by our left-branching complexity measure is shared with their word-by-word increases in high gamma for every word in a constituent. Our results complement their results in five ways: (1) by showing a functional asymmetry in neural processing of dependencies that go from the head to the dependent (i.e., left-branching) compared to the other way around; (2) by showing the spatial specificity of these effects with fMRI; (3) by a 15 fold increase in the number of participants to ensure robust effects; (4) by increasing the variability of the sentence structures used, since Nelson et al. (2017) restricted their sentence materials to only a single sentence structure; and finally (5) by extending these results from the visual modality only to the auditory modality as well, showing that the effects of left-branching complexity are likely to be supramodal. The absence of modality specific effects for the temporal dynamics analyses supports this conclusion. While it is easier to compare the increased response over the sentence to the oscillatory results discussed above, our results may also be compared with the results of Pallier et al. (2011). They found that fMRI responses got increasingly slower for larger constituent sizes in the more posterior LIFG pars triangularis, but not in the more anterior LIFG pars orbitalis (matching the posterior location of our LIFG finding; see Figure 4A), as well as for similar locations to ours in anterior and posterior superior temporal sulcus (pSTS). In addition, Regev et al. (2013) report highly similar time courses for spoken and written narratives in LIFG and pSTS (and additionally in precuneus and angular gyrus, regions that might be attributed to processing of their narrative stimuli). Below we interpret the results by focusing on the operations targeted by our left-branching complexity measure, using the effects of right-branching complexity for contrast and comparison.

A crucial aspect of language processing is the binding of the head of a verb phrase to its arguments. At least two factors determine the processing complexity of unifying a verb phrase. One is the position–distance between the head and its arguments in a sentence. The second is the order of heads and arguments. There are several possible reasons for this, such as an additional processing cost of storing unbounded arguments, as well as an asymmetry in predictive power (the head is a stronger prior for arguments than vice versa). In our study we computed a complexity measure for each individual sentence in the experiment, based on a formal characterization of the processing consequences of simultaneous unbounded arguments. This complexity measure modulated activity in LIFG and LpMTG in a parametric way, strongly suggesting that these regions together play a crucial role in the unification of lexical elements (i.e., words) into an overall structural sentence configuration.

When studying the activation dynamics across the sentence in the different nodes of the supramodal unification network, we observed a particularly interesting pattern of results in LIFG and LpMTG. In these regions, activity increased toward the end of the sentence for left-branching structures. No such effect was observed in LIPL (see Figure 4). We calculated the maximum point of simultaneous unification of unbounded arguments (usually referred to as dependents for other phrases, e.g., noun phrase, than verb phrases), which occurred on average around halfway into our sentences. Furthermore, the left-branching complexity measure indexes sentences with many simultaneous non-attached constituents, a cost which increases with each non-attached constituent presented, until a culmination point around halfway into the sentences. Since the priors for unification are relatively low in left-branching dependencies compared to right-branching dependencies, increased activation is expected toward the later parts of the sentence, given the increased number of unbounded arguments. In addition to this online processing complexity, new elements to the sentence structure are incrementally added and maintained. Hence the overall complexity of the output of online parsing operations is highest at the end of the sentence. This seems to be especially reflected in the IFG activation. An additional novel finding is that the complexity effect had an earlier time course in LpMTG than in LIFG. Both areas contribute to syntactic processing (Hagoort & Indefrey, 2014; Matchin & Wood, 2020). The earlier complexity effect in LpMTG might also include the consequences of the online processing complexity, next to the full sentence complexity that seems to dominate LIFG. Another possibility is that there are physiological delays, for example, in the metabolic consequences of neural activity that we observe through the hemodynamic response, that differ across brain regions. This is something to keep in mind when interpreting the result of the FIR analysis, which does not assume any particular shape of the HRF. Further exploration using additional modalities might be warranted: We note that recent meta-analytic results on written, spoken, and signed language suggested posterior LIFG in particular as an amodal hub (see Trettenbrein et al., 2021).

Right-branching complexity produced stronger activation than left-branching complexity in LaTL. This could be due to the stronger expectations about upcoming lexical-semantic information in the former case. This view on LaTL contribution to structure building fits well with the current views on the anterior temporal lobe function within linguistic and conceptual processing more generally (Patterson et al., 2007). There is, furthermore, evidence that conceptual combinations are processed in the anterior temporal lobe (e.g., Baron & Osherson, 2011). In line with our view, Pallier et al. (2011) only observed structure building (as indexed by an increasing response to increasing constituent size) in LaTL for materials with real words, and not in a Jabberwocky condition, where only posterior temporal and inferior frontal regions were observed (see also Goucha & Friederici, 2015).

The measure of simultaneous non-local dependencies that we used correlates with the presence of non-local dependencies as well as their total length. Our results thus show the neural underpinnings of the ubiquitous difficulty observed with processing non-adjacent dependencies (Gibson, 1998; Grodner & Gibson, 2005). In addition, our results reflect the increased difficulty of unifying complex left- compared to right-branching sentence aspects, at least in the context of a language like Dutch, which features considerable proportions of both. Our results suggest that *simultaneity* (or overlap) of multiple unresolved non-adjacent dependencies, rather than linear distance of non-adjacent dependencies, is a major factor contributing to the difficulty of processing non-adjacent dependencies (see additional analyses supporting this conclusion in the supplementary material). This highlights the importance of understanding which sentence processing operation(s) a complexity measure taps into. Previous literature indeed shows neurocognitive segregation of processing operations, drawing on computational models quantified as different (complexity) measures calculated on words (e.g., Bozic et al., 2010), sentences (e.g., Lopopolo et al., 2021), or narratives (e.g., Bhattasali et al., 2019; Willems et al., 2016).

In conclusion, we have characterized the basic spatiotemporal dynamics of a supramodal unification network consisting of the LIFG, bilateral MTG, and the LIPL. The finding that activity in this network increases over the sentence is novel (see possibly related oscillatory modulations in ECoG and MEG, respectively: Fedorenko et al., 2016; Lam et al., 2016). These results support our interpretation that binding of unbounded arguments increases toward the end of the sentence, resulting in stronger activations in LIFG and LpMTG. We also show the neural underpinnings of the ubiquitous difficulty observed with processing overlapping non-adjacent dependencies is independent of modality. Both listening to speech and reading activate the same neuronal circuitry once the modality-specific input is mapped onto lexical items, in the service of the brain’s capacity to make sense beyond the processing of single words.

## ACKNOWLEDGMENTS

We thank Laura Arendsen, Manuela Schuetze, Tineke de Haan, and Charlotte Poulisse for assisting with stimuli construction, participant recruitment, data collection, and preprocessing.

## FUNDING INFORMATION

This work was supported by the Max Planck Society. Julia Uddén, Riksbankens Jubileumsfond (https://dx.doi.org/10.13039/501100004472). Julia Uddén, Swedish Collegium of Advanced Studies, the Swedish Brain Foundation (Hjärnfonden postdoc grant, to J.U.). Peter Hagoort, the Spinoza Prize. Peter Hagoort, the Royal Netherlands Academy Professorship Prize. Peter Hagoort, the NWO Grant Language in Interaction, grant number 024.001.006.

## AUTHOR CONTRIBUTIONS

**Julia Uddén**: Conceptualization; Data curation; Formal analysis; Investigation; Methodology; Project administration; Resources; Software; Writing – original draft; Writing – review & editing. **Annika Hultén**: Conceptualization; Data curation; Investigation; Methodology; Project administration; Software. **Jan-Mathijs Schoffelen**: Resources. **Nietzsche Lam**: Data curation; Investigation; Project administration; Software. **Karin Harbusch**: Resources. **Antal van den Bosch**: Resources. **Gerard Kempen**: Resources. **Karl Magnus Petersson**: Conceptualization; Methodology; Resources. **Peter Hagoort**: Conceptualization; Methodology; Writing – original draft; Writing – review & editing.

## TECHNICAL TERMS

Unification: The process of combining word information, retrieved from memory, into representations of sentence-level meaning that are constructed on the fly.
Unification complexity or Left-branching complexity (of a sentence): The number of non-adjacent elements that have to be kept online, at the point in the sentence where most non-adjacent elements are simultaneously kept online (i.e., the maximum used stack depth).
Supramodal process: A process that is independent of input modality (e.g., independent of visual or auditory input modality).
