# Supplementary material for: Supramodal Sentence Processing in the Human Brain: fMRI Evidence for the Influence of Syntactic Complexity in More Than 200 Participants
Source: Neurobiol Lang (Camb). 2022 Sep 29;3(4):575–98. doi: 10.1162/nol_a_00076 (PMC10158636; doi:10.1162/nol_a_00076)
Supplement: Supplementary file 1 [file nol-3-4-575-s001.pdf]

S1. Supplementary methods

S1.1 Recording and post-processing of auditory stimuli

The audio files were recorded in stereo at 44100 Hz. During the post processing the audio files were low-pass filtered at 8500 Hz and normalized so that all audio files had the same peak amplitude, and same peak intensity. In the word-list condition, each word was separated by 300 ms of total silence. The transition from silence to speech was ramped at the onset (rise-time of 10 ms) and offset (fall-time of 10 ms) of single words in the word-list condition, and for sentence onset.

S1.2 Practice task

Prior to the task, participants read a written instruction of the task and asked questions for clarification. Furthermore, the experimenter emphasized that the sentences and word-lists should be attended carefully, and discouraged attempts to integrate the words in the word-list condition. Finally, to familiarize the participants with the task, they did a practice task with stimuli separate from the actual experiment.

S1.3 Preprocessing

Co-registration of the structural and functional images was checked for each individual subject by displaying the structural and the first functional image with SPM's checkreg. Normalization was checked by displaying the structural image, the functional image and the template, again using checkreg. No deviant co-registration or normalization was found.

S1.4 Left vs right-branching complexity

In order to compare the effects of left- and right-branching complexity directly, we analyzed a subset of the stimulus materials, selecting 320 of the 360 sentences, with their word-lists versions. We removed the sentences that had a combination of a high left-branching and a low right-branching complexity. In the resulting subset, the left- and right-branching complexity did not differ significantly over sentences (Wilcoxon test; ranksum: 103984, $Z = .64$, $P = .52$, thus balancing the stimulus set for the left- vs. right-branching comparisons). We performed comparisons of left- vs. right-branching complexity on this stimulus set, by creating the same model as described in the main methods section.

Similarly, in order to compare the dynamic effects for left and right-branching processing load (directly), we used the same methods as in section (2.6.1.1). This model was implemented in SPM12, without orthogonalization between pmods. Thus, the order of entering pmods does not matter.

S1.5 First level model: total dependency length

An identical model to that used to investigate the left and right-branching complexity was used, but instead of these measures, we now used the total dependency length measure.

S2. Supplementary results

S2.1 Left vs right-branching complexity

We analyzed the difference between the left and right-branching complexity directly. To do so, we selected a subset of the stimulus material, 320 of the 360 sentences, and their word-lists versions. In this subset, the left and right-branching complexity measures did not differ over sentences, as measured with a Wilcoxon rank test (rank sum: 103984, $Z = .64$, $P = .52$). When contrasting left > right-branching complexity, for sentences > word-lists, a significant effect was observed in the LIFG ROI. In addition, analyzing the sentences and word-lists separately, there was a left-branching > right-branching complexity effect for sentences in the LpMTG ROI. No corresponding effects where observed for right-branching > left-branching complexity, and there were no effects of left-branching > right-branching or right-branching > left-branching complexity in word-lists (see Table S1). Similarly, we tested the left-branching vs right-branching complexity effects in the dynamic changes across timebins (see Table S2). There was an effect for left-branching > right-branching complexity, for the increase across timebins, in the LpMTG locus, both when looking at sentences separately and when contrasting sentences to word lists.

Table S1

Supramodal left vs right-branching complexity.

Region	Cluster size	Cluster P_{FWE}	MNI- coordinates x	y	z	Voxel P_{FWE}	Voxel T_{201}
Left > Right branching*<br>Sentences > words							
ROI LIFG 10mm, SVC			-40	20	4	.045	2.88
Left > Right branching*,**<br>Sentences							
ROI LpMTG 10mm, SVC	59	.042					
			-52	-38	0	.036	3.00

No significant activations at whole brain or ROI-level for: Right, Sentences > words*; Right > Left*, Sentences > words; Right > Left*, Sentences; Right, Words > Sentences; Negative effect of Right, Sentences.

Note: * The direct comparisons of left vs right-branching complexity were performed on a subset of 320 (out of 360) sentences. In this subset, the left and right-branching complexity measures did not differ significantly over sentences ($p > .50$). SVC: small volume correction

**see supplementary methods for significant visual > auditory or auditory > visual activations.

Table S2

Supramodal left- vs right-branching complexity, *increase* across time-bins.

Region	Cluster size	Cluster P_{FWE}	MNI- coordinates x	y	z	Voxel P_{FWE}	Voxel T_{201}
Left > Right branching* Sentences > words							
ROI LpMTG 10mm, SVC	83	.027					
			-46	-42	2	.011	3.63
Left > Right branching*							
Sentences	92	.022					
			-46	-42	2	.010	3.70

No significant activations (neither at ROI-level) for: Any of the corresponding decreases; Right > Left*, Sentences > words, Right > Left*, Sentences. Furthermore, there were no effects of modality (visual > auditory or auditory > visual).

Note: *The direct comparisons of left vs right-branching complexity were performed on a subset of 320 (out of 360) sentences, turning off orthogonalization between pmods in SPM12. In this subset, the left and right-branching complexity measures did not differ significantly over sentences (p > .50). SVC: small volume correction

Table S3

Supramodal effect of Sentences vs low level baseline (fixation/rest).

Region	Cluster size	Cluster p_{FWE}	MNI- coordinates x	y	z	Voxel p_{FWE}	Voxel T_{201}
Sentences > IBI							
LpMTG/LaTL/LIFG/LIPL/Left Fusiform G	7265	<.001					
LMTG (mid)			-56	-10	-12	<.001	16.21
LpMTG/STS			-54	-46	10	<.001	15.75
LaTL			-48	14	-22	<.001	14.64
Left Fusiform G I			-36	-38	-18	<.001	7.85
Left Fusiform G II			-30	-34	-16	<.001	7.67
Thalamus			-8	-28	-2	<.001	7.53
LIFG (BA 45)			-56	28	8	<.001	7.52
LIFG (BA 47)			-40	32	-10	<.001	6.94
L Medial TL I			-40	-16	-20	0.002	5.33
L Medial TL II			-22	-14	-14	0.014	4.86
+ *LIPL*							
RpMTG/RaSTG	2909	<.001					
RaSTG			48	14	-20	<.001	11.92
RMTG (mid)			54	-6	-14	<.001	11.73
RpMTG/STS			50	-36	6	<.001	7.66
R Rolandic Operculum			40	-20	20	0.037	4.61
R Fusiform G	1075	.012					
R Fusiform G posterior			30	-52	-10	.001	5.50
R Fusiform G anterior			28	-30	-18	.001	5.49
Left orbitofrontal gyrus			-6	36	-18	<.001	7.15
LSFG			-10	58	32	<.001	6.29
Left precentral gyrus			-42	-6	48	<.001	5.76

S2.2 Modality specific effects: Left vs. right-branching complexity

Here we report modality specific effects (visual > auditory) and (auditory > visual) effects that were present in some of the contrasts reported in results sections 3.2 and forward. There were visual > auditory effects when comparing left > right-branching complexity, for sentences > word-lists, (Figure S1, Table S4). In a follow up analysis, we tested whether the observed effects in these visual > auditory contrasts were due to an effect of left-branching complexity in the opposite/negative direction (i.e. higher BOLD response for lower complexity) for the auditory group. This was the case (Table S4).

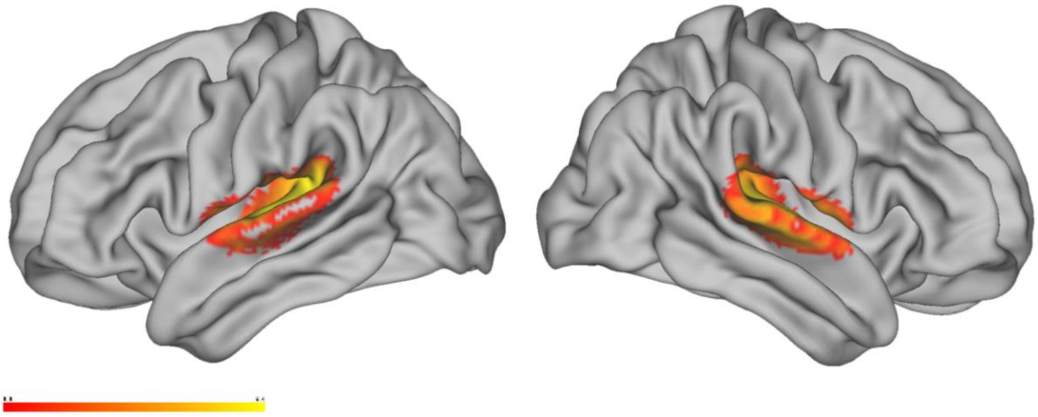

Figure S1. Left > Right, Sentences > Word-lists. Visual > Auditory (*P* < .005 uncorrected).

Table S4

Modality specific effects left (vs right) complexity in sentences vs word-lists

Region	Cluster size	Cluster P_{FWE}	MNI- coordinates x	y	z	Voxel P_{FWE}	Voxel T_{201}
Left vs right-branching<br>Sentences > word-lists<br>Visual > Auditory							
LSTG	2954	<.001					
			-50	-18	4	<.001	7.32
			-40	-30	10	<.001	6.38
RSTG	2429	.001					

			54	-18	6	<.001	6.57
			56	-8	2	<.001	6.24

No significant activations for visual > auditory or auditory > visual in: Left, sentences > word-lists; Right, sentences; Left vs right, sentences. No significant activations for auditory > visual in: Left, Sentences; Left vs right, sentences > word-lists. No significant activations for visual > auditory in: Right, sentences > word-lists.

Table S5

Modality specific auditory effect of left (vs right) branching complexity.

Region	Cluster size	Cluster P_{FWE}	MNI- coordinates x	y	z	Voxel P_{FWE}	Voxel T_{201}
Left > right-branching<br>Negative direction<br>Sentences > word-lists<br>Auditory							
LSTG/Heschl's gyrus	5217	<.001					
			-50	-20	6	<.001	9.18
			-40	-30	10	<.001	7.74
RSTG	3131	<.001					
			-54	-18	6	<.001	8.05

S2.2 The total dependency length complexity measure

The left-branching complexity measure is each sentence's maximum number of simultaneously open left-branching dependencies. For comparison of the complexity measures we use with the total dependency length measure (Futrell et al. 2015). A universal tendency for minimizing total dependency length was recently reported (Futrell *et al.* 2015). We calculated the total dependency length for our sentences. Using partial correlations controlling for number of letters, words and syllables, the total dependency length measure was robustly correlated with our left-branching complexity measure ($\rho_{(rho)}$ = .58, P < .001), but not with right-branching complexity measure ($\rho_{(rho)}$ = -.03, P = .52). We analyzed the total dependency length in a separate model, assessing effects of neural infrastructure subserving processes of maintenance

of lexical items, as a complement to the analysis of left-branching complexity. We observed no significant effect of total dependency lengths in the sentences > word-lists comparison. However, we observed an effect of increasing total dependency length in the LpMTG ROI, for the separate sentence contrast (Figure S2 and Table S6). There were modality specific effects, in the direction of auditory > visual (see Figure S3 and Table S7). We followed up by analyzing the visual and auditory groups separately, the auditory sample had significant clusters for total dependency length, in both sentences > word-lists and sentences when analyzed separately (Figure S3 and Table S7).

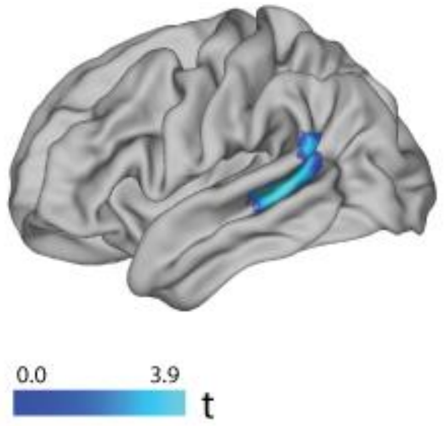

Figure S2. Positive parametric effect of total dependency length in sentences, $P < .005$ uncorrected. There were significant clusters and voxels in the LpMTG ROI. Conjuction over auditory and visual groups.

Table S6

Supramodal effect of total dependency length (TDL) in sentences and word-lists.

Region	Cluster size	Cluster p_{FWE}	MNI- coordinates x	y	z	Voxel p_{FWE}	Voxel T_{201}
TDL, Sentences							
ROI LpMTG 10mm, SVC	279	.011					
ROI LpMTG 10mm, SVC			-50	-40	2	.001	4.13
No significant activations (neither at ROI-level) for TDL, Sentences > words							

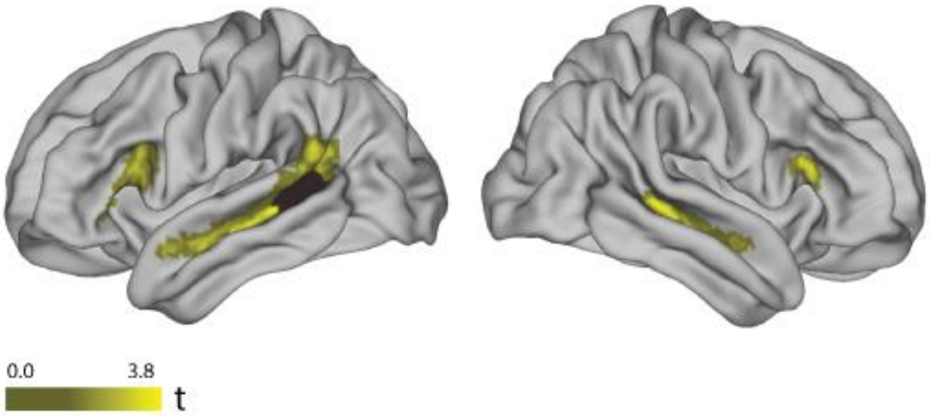

Figure S3. Positive parametric effect of total dependency length in sentences > word lists, Auditory sample, $P < .005$ uncorrected. In the LpMTG ROI, there was a significant Auditory > Visual effect, for sentences > word lists.

In discussion of these results, we note that our left-branching complexity measure, which targets overlapping non-adjacent dependencies, resulted in a more robust effect than the subtler effects observed using the (correlated) total dependency length measure. This pattern of results suggests that simultaneity (or overlap) of multiple unresolved non-adjacent dependencies, probes a partly different aspect of sentence processing than linear distance of non-adjacent dependencies (as indexed by the TDL measure). Both processes probably contribute to the difficulty of processing non-adjacent dependencies, but the simultaneity had the greater effect, at least on the BOLD-response. However, in most cases, these factors will be correlated, so the existing observations on difficulty associated linear distance (not controlling for simultaneity and direction of those dependencies) in the literature, are expected.

Table S7

Modality specific effects of total dependency length (TDL).

Region	Cluster size	Cluster $p_{FWE-corr}$	MNI- coordinates x	y	z	Voxel $p_{FWE-corr}$	Voxel T_{201}
TDL, Auditory > Visual Sentences > word-lists							
ROI LpMTG 10mm, SVC			-50	-42	-4	.036	2.99
TDL, Auditory Sentences > word-lists							
LpMTG/STS	1281	.021					

ROI LIFG, 10mm, SVC	121	.025	-48	16	18	.010	3.44
TDL, Auditory > Visual Sentences							
LSTG	1652	.006					
RSTG	1518	.009					
TDL, Auditory Sentences							
LpMTG	3739	.000					
			-52	-38	4	.000	6.55
			-54	-12	-4	.002	5.32
RpMTG	2662	.000					
			52	-16	-8	.002	5.24
			50	-34	2	.009	4.90

No significant activations (neither at ROI-level) for: TDL, Sentences > words, Visual > Auditory; TDL, Sentences, Visual > Auditory
